# Supplementary material for: Spatial and Ecological Drivers of Genetic Structure in Greek Populations of Alkanna tinctoria (Boraginaceae), a Polyploid Medicinal Herb
Source: Front Plant Sci. 2021 Jul 14;12:706574. doi: 10.3389/fpls.2021.706574 (PMC8317432; doi:10.3389/fpls.2021.706574)
Supplement: Supplementary file 1 [file Data_Sheet_1.pdf]

Supplemental Information for:

Spatial and ecological drivers of genetic structure in Greek populations of *Alkanna tinctoria* (Boraginaceae), a polyploid medicinal herb

Muhammad Ahmad, Thibault Leroy, Nikos Krigas, Eva Temsch, Hanna Weiss-Schneeweiss, Christian Lexer, Eva Maria Sehr, and Ovidiu Paun

Table of Contents:

**Table S1**	Page 2
**Table S2**	Page 3
**Table S3**	Page 4
**Figure S1**	Page 5
**Figure S2**	Page 6
**Figure S3**	Page 7
**Figure S4**	Page 8
**Figure S5**	Page 9
**Notes S1**	Page 10

Table S1. Acronym, number of individuals included in the RAD-seq analyses (N), voucher and/or living material information with IPEN (International Plant Exchange Network) accession number, and geographic coordinates of localities included in this study.

Acronym	Greek Region	N	Voucher	Latitude	Longitude
Alkanna sieberi					
AT28	Central Crete	11	GR-1-BBGK-19,668	34.92904	24.77959
AT27	Western Crete	11	GR-1-BBGK-19,667	35.18339	24.24294
Alkanna tinctoria					
AT01	South mainland	9	GR-1-BBGK-18,6127	37.97558	23.62006
AT10	South mainland	8	GR-1-BBGK-18,6136	37.87581	23.77331
AT02	South mainland	9	GR-1-BBGK-18,6128	37.96651	23.77741
AT06	South mainland	9	GR-1-BBGK-18,6133	38.03168	23.49035
AT07	South mainland	9	GR-1-BBGK-18,6134	37.94776	22.97474
AT08	South mainland	9	GR-1-BBGK-18,6134	37.91818	22.9967
AT17	Central mainland	9	GR-1-BBGK-19,509	39.35058	22.97063
AT19	Central mainland	9	GR-1-BBGK-19,658	38.94382	22.86016
AT21	Central mainland	9	GR-1-BBGK-19,660	38.79034	22.44321
AT25	Central mainland	9	GR-1-BBGK-19,665	38.50414	23.06436
AT03	North mainland	11	GR-1-BBGK-18,6081	40.63138	22.97166
AT04	North mainland	9	GR-1-BBGK-18,6091	40.64277	22.99777
AT13	North mainland	8	N/A	40.64666	22.98777
ATA8	North mainland	9	GR-1-BBGK-18,6100	40.11294	23.31472

Table S2. Information about various inputs used in downstream analysis of RAD-seq data

Analysis/Software	missingness	Number of SNPs	Figure/Table	Comment
TESS3	10%	7,935	Figure 1A, S2A & S3A	1 SNP/locus
STRUCTURE	10%	1,000	Figure 1B, S2B & S3A	1 SNP/locus
Genotype frequency vs allele frequency	0%	16,107	Figure 2	
Pairwise relatedness	0%	16,107	Figure 3A	
Principal component analysis	0%	16,107	Figure 3B	
Gradient forest	0%	16,107	Figure 4A	
FST for IBD and IBE	0%	16,107	Figure 4B & 4C	
Genomic diversity	0%	16,107	Table 2	
Multiple matrix regression	0%	16,107	Table 3	
BayPass	0%	16,107	Figure S4	

Table S3. Pairwise F_{ST} among sampling localities of *Alkanna tinctoria* and *A. sieberi*. F_{ST} was estimated from 16,107 SNPs of RAD-seq data present across all 148 individuals. The highest and lowest F_{ST} estimates in *A. tinctoria* are highlighted in bold.

	A. sieberi		***A. tinctoria***												
	Crete		Southern Greece						Central Greece				Northern Greece		
	AT28	AT27	AT01	AT10	AT02	AT06	AT07	AT08	AT17	AT19	AT21	AT25	AT03	AT04	AT13
AT28															
AT27	0.333														
AT01	0.315	0.178													
AT10	0.342	0.174	0.025												
AT02	0.341	0.198	0.029	0.035											
AT06	0.332	0.180	0.017	0.032	0.032										
AT07	0.328	0.182	0.052	0.074	0.066	0.050									
AT08	0.348	0.199	0.075	0.089	0.095	0.073	0.032								
AT17	0.361	0.229	0.097	0.114	0.118	0.098	0.120	0.123							
AT19	0.373	0.233	0.102	0.116	0.124	0.104	0.123	0.126	0.062						
AT21	0.386	0.252	0.130	0.144	0.155	0.132	0.148	0.149	0.092	0.083					
AT25	0.409	0.279	0.152	0.172	0.174	0.151	0.175	0.181	0.144	0.150	0.174				
AT03	0.309	0.240	0.150	0.180	0.156	0.161	0.146	0.186	0.189	0.213	0.226	0.255			
AT04	0.310	0.238	0.142	0.173	0.152	0.155	0.143	0.176	0.174	0.198	0.212	0.242	0.010		
AT13	0.325	0.257	0.152	0.189	0.160	0.167	0.150	0.193	0.199	0.223	0.238	0.269	**0.007**	0.010	
ATA8	0.338	0.268	0.162	0.198	0.166	0.171	0.163	0.205	0.210	0.232	0.251	**0.280**	0.071	0.077	0.070

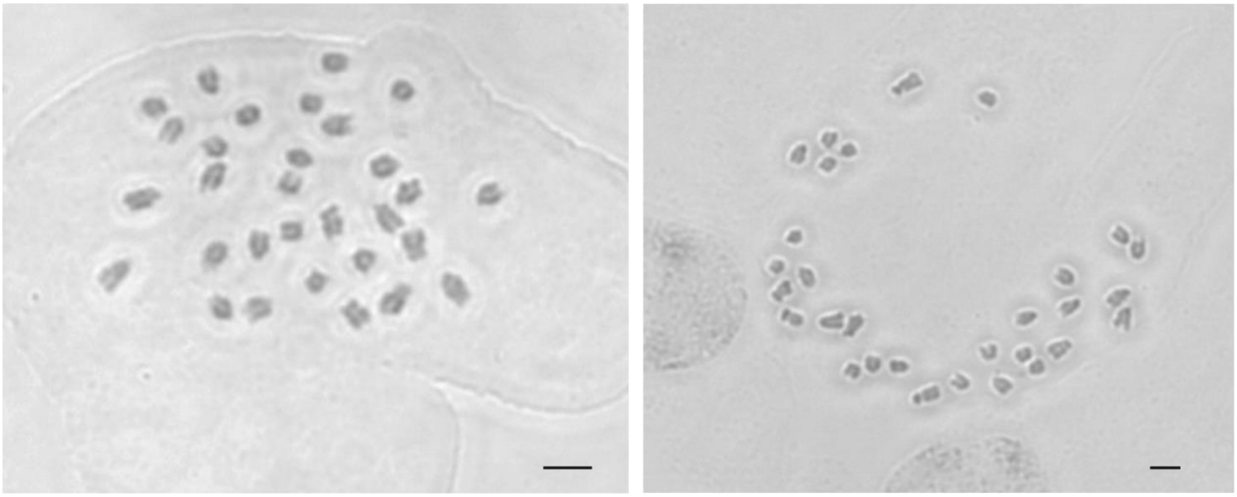

Figure S1. Chromosomes of *Alkanna tinctoria* (2*n* = 30). Scale bar 5µm. Roots of two individuals of *A. tinctoria* collected from AT03 locality were used for chromosomal counts.

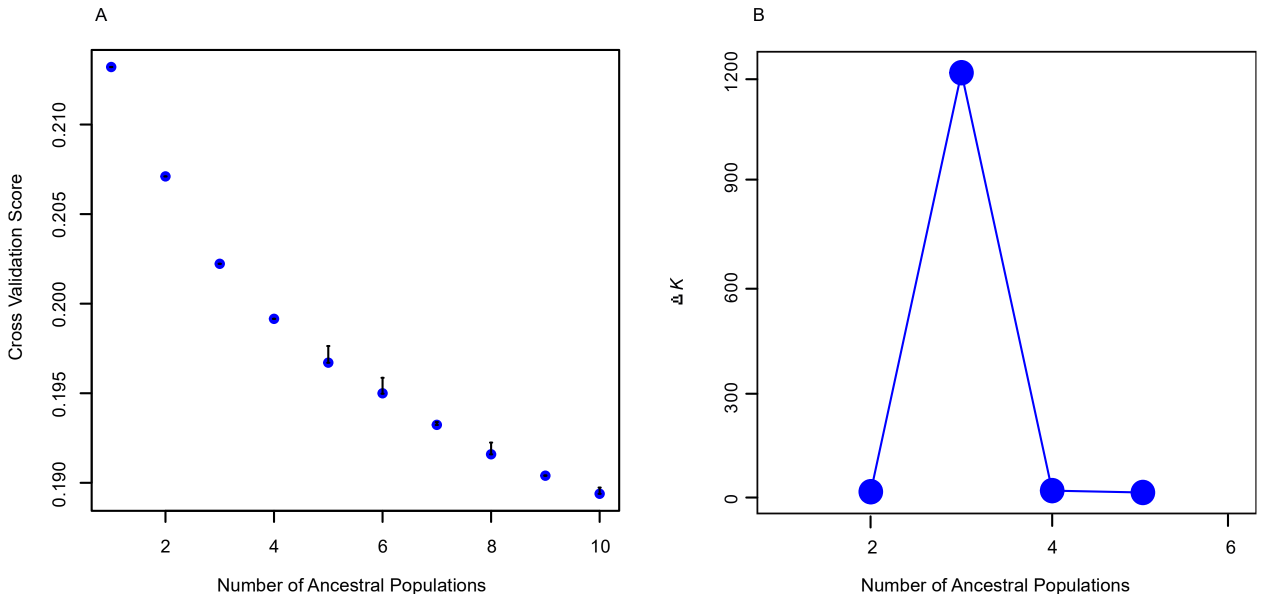

Figure S2. Cross validation score (A) and *ΔK* (B) showing optimal *K* identified by TESS3 and STRUCTURE HARVESTER, respectively. In the cross validation plot, the largest steps can be seen between *K1* and *K3*. After *K3*, the values of cross validation score decreased at a slower rate.

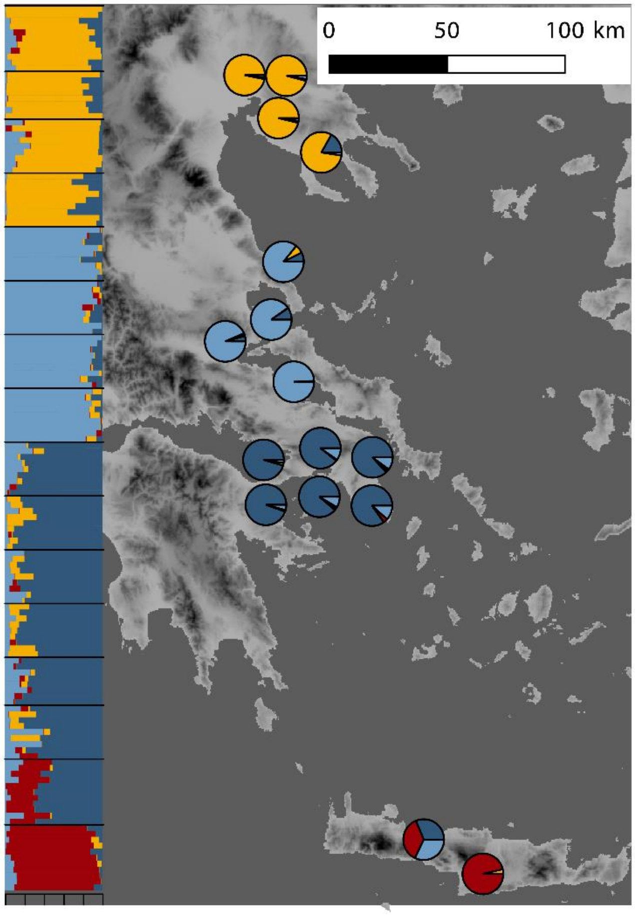

Figure S3. Genetic structure in studied *Alkanna* populations inferred based on EBG-derived tetraploid genotypes (n = 148). Ancestry proportions inferred with TESS3 (7,935 SNPs; 10% missingness) averaged for sampling locality are plotted as pie charts. Inset shows ancestry proportions from STRUCTURE (1,000 SNPs; 10% missingness) as vertical bars where each vertical bar represents an individual. Each color represents a genetic cluster. Ancestry proportions based on *K4*. The map layer of Greece was extracted from elevation data obtained from the WorldClim database (Hijmans et al., 2005).

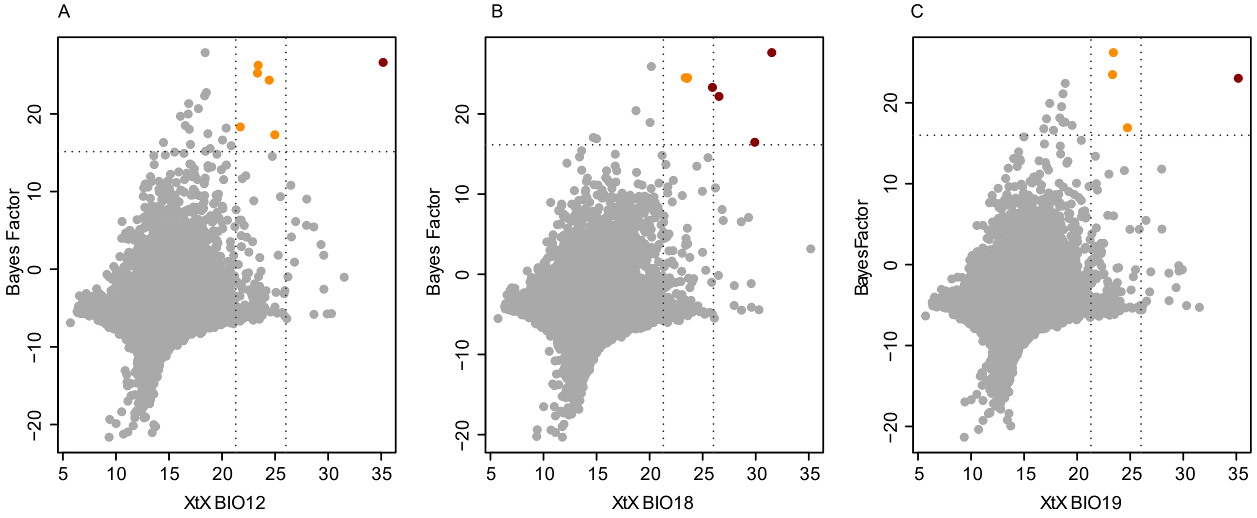

Figure S4. Outlier analysis of genotype-environment association (BayPass) of *Alkanna tinctoria* based on 16,107 SNPs derived from RAD-seq data**.** Scatter plots of correlation of XtX and Bayes factor (BF) for SNPs showing association to environmental variables. (A) BIO12, Annual Precipitation (B) BIO18, Precipitation of Warmest Quarter and (C) BIO19, Precipitation of Coldest Quarter. Scatter dots highlighted in different colors represent associated SNPs at different thresholds. Red with XtX and BF > 99.9%; orange with XtX > 99% and BF > 99.9%.

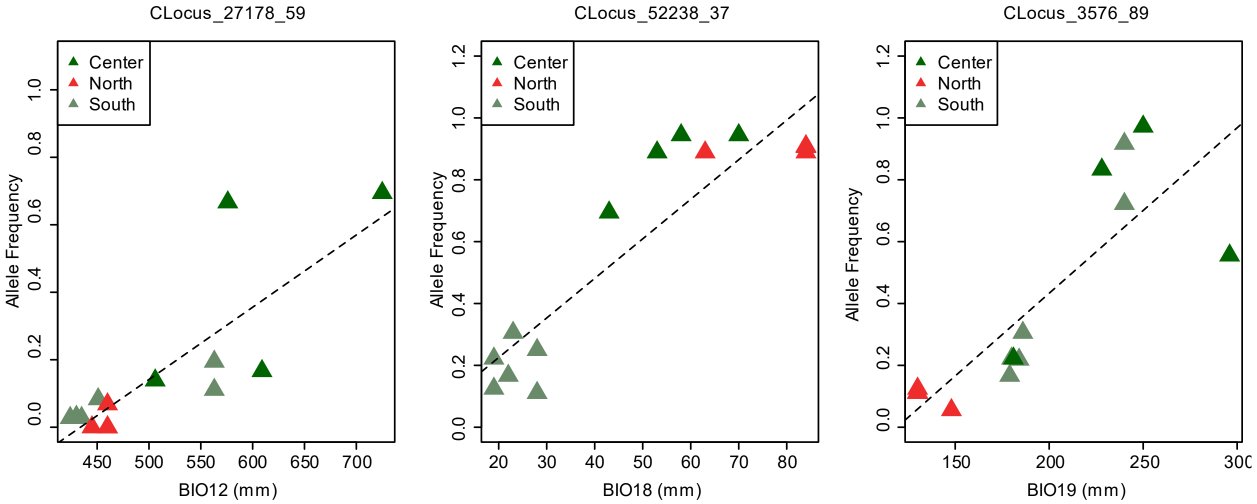

Figure S5: Changes in allele frequencies of loci identified by BayPass along the environmental gradient (BIO12 = Annual Precipitation, BIO18 = Precipitation of Warmest Quarter and BIO19 = Precipitation of Coldest Quarter).

Notes S1

```
#######################################################
###Custom function used to calculate pi from EBG outputfile###
#######################################################
pi_calculator <- function(df, ploidy, total_sites){  #total length is number of loci* bp per locus
  df[df == -9] <- NA
  j <- rowSums(df, na.rm = T)
  n <- ((ncol(df)-rowSums(is.na(df)))*ploidy)
  a <- 2*j*(n-j)
  b <- n*(n-1)
  local_pi <- (a/b)
  sum_local_pi <- sum(local_pi)
  total_pi <- sum_local_pi/total_sites
  return(total_pi)
}

###import your data of EBG output with rows as individuals and columns as counts of alternate
#alleles
#example of run
poly <- data.frame(read.table("input.txt",sep="\t", header=T))
total_sites = 2446
ploidy = 4
###subsetting the data for populations
> AT28 = poly[,2:12]
#calculating pie
> pi_calculator(AT28, 4, 2446*94)
0.007107616


####################################
###script for random selection of SNPs###
####################################
#!/bin/bash

# Author: Livio Antonielli (livio.antonielli@ait.ac.at)

# Provide data
echo -e "\n==================== Importing data ====================\n"

read -p 'Provide the absolute path of "Ind" file (i.e. first column with sample names): ' Ind
read -p 'Provide the absolute path of "Pop" file (i.e. second column): ' Pop
read -p 'Provide the absolute path of the variable file (i.e. the main table): ' output
read -p 'Type the number of columns to keep (random selection): ' num
read -p 'Provide the absolute path of "shuffle.awk" script: ' shuffle
```

```
# DOS to Unix conversion
echo -e "\n==================== Windows to Unix file conversion
====================\n"

awk '{ sub("\r$", ""); print }' $Ind > Ind_unix.temp
awk '{ sub("\r$", ""); print }' $Pop > Pop_unix.temp


# Selecting n random columns from main variable table
echo -e "\n==================== Selecting random columns
====================\n"

cat $output | awk -f $shuffle -v ncols=$num > random_table.temp
seq -s' ' $num | sed 's/ / V/g; s/^/V/' > random_head.temp
cat random_head.temp random_table.temp > var.temp

# Building the final table
echo -e "\n==================== Generating the final table ====================\n"

paste Ind_unix.temp Pop_unix.temp var.temp | tr ' ' \\t > table.temp

body() {
   IFS= read -r header
   printf '%s\n' "$header"
   "$@"
}

cat table.temp | body sort -k2 -V > table_ord.temp

echo "//configuration" > head1.temp
echo "//#alleledigits(1~4)	#outputdigits(~1)	#missingallele #ambiguousallele
	#nthreads(1~64)" > head2.temp
echo "1	8	0	9	16" > head3.temp
echo "//genotype" > head4.temp
echo "//end of file" > tail.temp

cat head*.temp table_ord.temp tail.temp > output_table.txt

rm -rf *.temp

# End
echo -e "\n==================== The table is ready! ====================\n"
```

```
##############################
###Random selection of columns###
##############################

# https://stackoverflow.com/questions/36187942/random-selection-of-columns-using-linux-command###

function shuffle(a,n,k) {
    for(i=1;i<=k;i++) {
      j=int(rand()*(n-i))+i
      if(j in a) a[i]=a[j]
      else a[i]=j
      a[j]=i;
    }
  }

  BEGIN{srand(); shuffle(ar,NF,ncols)}
       {for(i=1;i<=ncols;i++) printf "%s", $(ar[i]) FS; print ""}
```
